# Comprehensive Assessment of Combatants’ Psychological and Psychophysiological State in Exposure Therapy of Post-Traumatic Stress Disorder Using Virtual Reality

**DOI:** 10.17691/stm2024.16.5.04

**Published:** 2024-10-30

**Authors:** L.N. Kasimova, A.N. Kuznetsov, I.I. Kropinova, D.V. Kuznetsov, M.G. Volovik, M.V. Svyatogor, E.M. Sychugov, G.Y. Borovskoy, M.E. Khalak

**Affiliations:** MD, DSc, Professor, Head of Psychiatry Department; Privolzhsky Research Medical University, 10/1 Minin and Pozharsky Square, Nizhny Novgorod, 603005, Russia; Head of Immersive Technologies and Remote Rehabilitation Laboratory; Privolzhsky Research Medical University, 10/1 Minin and Pozharsky Square, Nizhny Novgorod, 603005, Russia; Research Assistant, Immersive Technologies and Remote Rehabilitation Laboratory; Privolzhsky Research Medical University, 10/1 Minin and Pozharsky Square, Nizhny Novgorod, 603005, Russia; Master’s Degree Student, Psychophysiology Department, Faculty of Social Sciences; National Research Lobachevsky State University of Nizhny Novgorod, 23 Prospekt Gagarina, Nizhny Novgorod, 603022, Russia; Research Assistant, Immersive Technologies and Remote Rehabilitation Laboratory; Privolzhsky Research Medical University, 10/1 Minin and Pozharsky Square, Nizhny Novgorod, 603005, Russia; Master’s Degree Student, Psychophysiology Department, Faculty of Social Sciences; National Research Lobachevsky State University of Nizhny Novgorod, 23 Prospekt Gagarina, Nizhny Novgorod, 603022, Russia; DSc, Leading Researcher, University Clinic; Privolzhsky Research Medical University, 10/1 Minin and Pozharsky Square, Nizhny Novgorod, 603005, Russia; PhD, Associate Professor, Psychiatry Department; Privolzhsky Research Medical University, 10/1 Minin and Pozharsky Square, Nizhny Novgorod, 603005, Russia; Teaching Assistant, Psychiatry Department; Privolzhsky Research Medical University, 10/1 Minin and Pozharsky Square, Nizhny Novgorod, 603005, Russia; Senior Laboratory Technician, Psychiatry Department; Privolzhsky Research Medical University, 10/1 Minin and Pozharsky Square, Nizhny Novgorod, 603005, Russia; PhD, Associate Professor, General and Clinical Psychology Department; Privolzhsky Research Medical University, 10/1 Minin and Pozharsky Square, Nizhny Novgorod, 603005, Russia

**Keywords:** post-traumatic stress disorder, combatants, virtual reality, virtual reality exposure therapy, heart rate variability, stress markers

## Abstract

**The aim of the study** was to develop the rehabilitation technology that enables the monitoring of psychophysiological stress markers during exposure therapy of post-traumatic stress disorder in combatants using virtual reality (VR).

**Materials and Methods:**

The study involved 69 men: 31 combatants (mean age — 35.61±9.13 years) and 38 healthy research subjects — a control group (mean age — 24.68±5.71 years) not engaged in active combat.

Post-traumatic stress disorder was diagnosed using Structured Clinical Interview for DSM and Mississippi Scale.

We suggested an original hardware and software system for exposure therapy in VR. Stimulus material included a number of virtual scenes: three combat scenes and a non-combat one.

Heart rate variability was recorded to control a patient’s state during the session. As markers of stressogenic situations we used CS-index (suggested by S.V. Bozhokin), as well as the indices of functional reserve and tension degree of the regulatory systems (according to R.M. Baevskiy).

**Results:**

There were revealed three main responses on VR scenes with an original content. The three variants were called as follows: “anxious”, “neutral”, and “inverse”. The suggested methodology enables to continuously monitor psychophysiological parameters during a certain session, and analyze their dynamics within a therapy course.

Using calculated indicators by Baevsky makes it possible to classify combatants by adaptive potential at the beginning and at the end of the exposure therapy course in VR; make use of online-control of a patient’s functional state in virtual environment, and create conditions for controlled information influence (CS-index by Bozhokin).

The preliminary results presented in the study are promising regarding the possibility to choose a personalized program of rehabilitation measures for each response type using a developed hardware and software system. Biological feedback on heart rate variability included in hardware and software system will contribute to train and harness a patient’s habit of an operative independent correction of the patient’s state.

## Introduction

One of promising directions in rehabilitation therapy is virtual reality exposure therapy (VRET). The technique is used in a complex rehabilitation of patients with mental disorders, in particular, to treat post-traumatic stress disorders (PTSD) [1]. Under the conditions of an increasing number of local military conflicts, VRET applicability for PTSD diagnosis and treatment in combatants is growing [2]. Virtual reality (VR) simulation of icant events causing a combat mental trauma enables to achieve patient’s high-level motivation and involvement in a rehabilitation process. It promotes successful desensitization including difficult cases, when the previous therapy failed to have any improvement [3].

However, the content self-demonstrated in virtual environment can induce the deterioration in emotional and psychophysiological state. Therefore, one of the key objectives when using VR technologies is the necessity to continuously monitor a patient’s functional state [4, 5].

Heart rate variability (HRV) is increasingly being used as a tool to assess the autonomic regulation dynamics in a real-time mode. The research by Polevaya et al. [6] proved the reliability and efficiency of the developed technique of event-related heart rate telemetry — an information-telecommunication technology for remote monitoring of stressful situations in terms of natural activity. The moment acute stress occurs is automatically determined by HRV total power (TP) decrease, while sympatho-vagal balance index (LF/HF) dramatically increases [7]. The relationship grounding is based on a triple-component theory of stress neurochemical mechanisms represented by Parin [8].

Continuous HRV recording technology consisting in efficient data collection and analysis [9] enables to early detect the conditions, which require immediate intervention. HRV data reflecting autonomic nervous system state make it possible to estimate body adaptive resources and reveal the risk of chronic diseases [10].

Real-time integrated analysis of recorded data (in particular, spectral and statistical HRV indices) contributes to detecting specific early biomarkers of extreme states, consequently providing controlled stimulus presentation during VRET. Objective measurement of “immersion” degree by functional state monitoring is the key to establish a patient’s individual tolerance window. Recorded heart rate parameters enable to timely provide a patient with the feedback on tension degree of his regulatory systems. Owing to the information, a patient can for some time decrease the tension switching to physical or some other activity [11]. Automated regulation of triggers intensity using biological feedback optimizes a therapeutic process improving the treatment efficiency and decreasing the risk of potential disturbances or non-adaptive reactions in the patients of this group.

The autonomic regulation dynamics in VRET can be assessed both: during the certain session, and throughout the rehabilitation program (at its different stages) in PTSD patients. Obviously, the detailed analysis of patients’ characteristics of the response on VR-scenes, as well as the fuller appreciation of their state will enable to develop personalized rehabilitation programs.

**The aim of the study** was to develop the rehabilitation technology enabling to exert control over psychophysiological stress markers during exposure therapy of post-traumatic stress disorder in combatants using virtual reality.

## Materials and Methods

### Sampling

The study involved 69 men: 31 combatants (mean age — 35.61±9.13 years) and 38 healthy research subjects (mean age — 24.68±5.71 years) not engaged in active combat — a control group.

The study was approved by the Ethics Committee of Privolzhsky Research Medical University, and carried out in accordance with the Declaration of Helsinki (2013). All participants gave their consent to be involved in the present study.

Combatants self-completed the questionnaire including socio-demographic characteristics (gender, age, education, marital status, etc.) and the information on their battle experience.

PTSD was diagnosed using a Structured Clinical Interview for DSM (SCID) and Mississippi Scale to determine the intensity of adaptive disorders due to post-traumatic stress resulted from engagement in active combat.

Depression was diagnosed using Beck Depression Inventory; and suicide risk was assessed by Suicide Behavior Questionnaire (SBQ).

10 combatants were diagnosed with PTSD according to SCID; the total score according to Mississippi questionnaire in this group was over 112 confirming the presence of marked adaptive disorders due to combat PTSD. 8 PTSD combatants were diagnosed with depression (the score according to Beck scale was ≥10), and 3 combatants were found to have a high suicide behavior risk.

### Hardware and software system

The developed prototype of hardware and software system included a VR-helmet HTC Vive Focus 3 (HTC Corporation, Taiwan), a wireless sensor for continuous HRV recording Callibri (Neurotech, Russia), a portable personal computer (basic characteristics: Core i7 12700H, 16Gb, NVIDIA GeForce RTX3070Ti 8Gb). The stimulus material included a number of virtual scenes developed on the basis of game engine Unreal Engine 5: three combat scenes and a non-combat scene. Using an original hardware and software system, the stimulus material was demonstrated on the computer screen and at the same time — was transmitted to a VR-helmet. In addition, the software supported HRV data collection from the sensor Callibri and the data display on an operator’s screen in real-time mode in the form of HRV indices diagram: the heart rate and the dynamic tension index of the cardiovascular system [12]. The diagrams were used to control the stress level in a research subject, as well as to correct the transmitted stimulus material parameters.

### Techniques

HRV indices were continuously recorded to estimate autonomic regulation dynamics throughout the session [9]. Before the investigation we determined the functional state at rest in the supine position within 5 min [13] followed by an orthostatic test [14]. After that a research subject was taught diaphragm breathing, and carried out a breathing test for 2 min [15]. The subjects were instructed in using the respiratory pattern in virtual reality in case they have subjective distress experience. Each research subject had a VR-helmet on the head; through the helmet a subject could observe the sequential demonstration of three battle scenes alternated with a non-combat (conditionally “relaxation”) scene.

## Results and Discussion

To study combatants’ response to VR-scenes, we used the following characteristics from HRV indices: functional reserve (FR) and tension degree (TD) of the regulatory systems; and to assess the functional state we used a phase plane with coordinates FR and TD.

**Figure 1.**
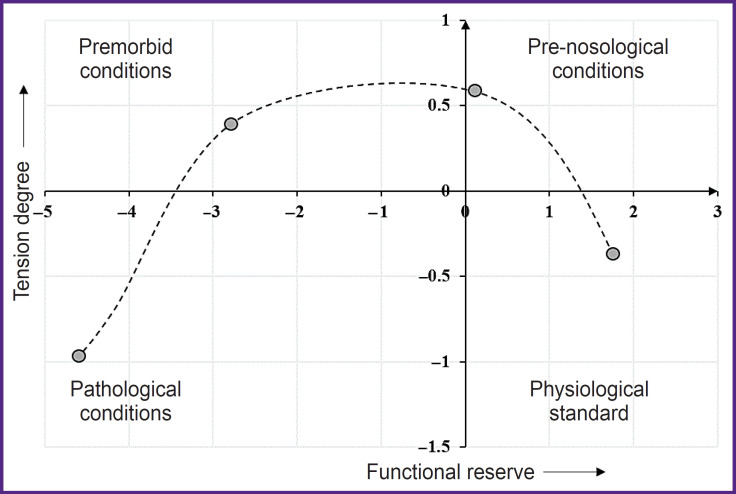
R.M. Baevskiy scheme [1]: the space of functional conditions in coordinates of functional reserve and tension degree of the regulatory systems (phase plane with the coordinates of functional reserve and tension degree, and four state classes)

According to Baevskiy classification [10], there are four classes of states: physiological standard, pre-nosological conditions, premorbid conditions, and pathological conditions (Figure 1).

In combatants with PTSD diagnosis made at the study entry, HRV dynamics indicated marked tension of regulatory systems and significant FR decrease: such condition was estimated as pre-nosological or premorbid.

According to our assessment of autonomic regulation, combatants’ functional state dynamics compared to the controls can be conditionally distinguished into three types.

**Figure 2.**
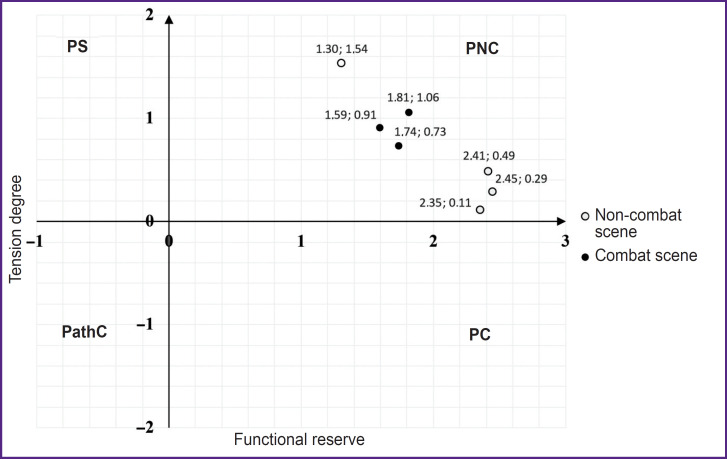
Functional state and physiological reserves dynamics when VR-content is demonstrated to a typical representative of an “anxious” type group (Patient 27 ID) Number near each point corresponds to its coordinates. PS — physiological standard, PNC — pre-nosological conditions, PC — premorbid conditions, PathC — pathological conditions

The combatants with the functional state deteriorating when being demonstrated combat scenes were referred to the first type. The group representatives were found to have distinct TD differentiation between combat and non-combat scenes: in the former case the index increased (functional state deterioration), and in the latter — the index decreased (functional state improvement). In a number of cases a deterioration response could occur when a non-combat scene was demonstrated; it can be explained by a late-onset manifestation related to the effect of the previous scenario containing battle actions. The conditional name of the group is “anxious” type reaction (Figure 2).

**Figure 3.**
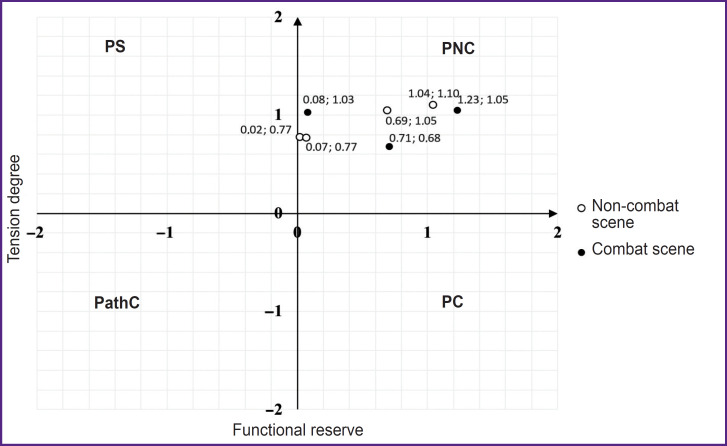
Functional state and physiological reserves dynamics when VR-content is demonstrated to a typical representative of a “neutral” type group (Patient 31 ID) PS — physiological standard, PNC — pre-nosological conditions, PC — premorbid conditions, PathC — pathological conditions

The second type was characterized by no changes and no distinct differentiation of the functional state when being demonstrated in VR both — combat and non-combat scenes. The conditional name of the group is “neutral” type response (Figure 3).

**Figure 4.**
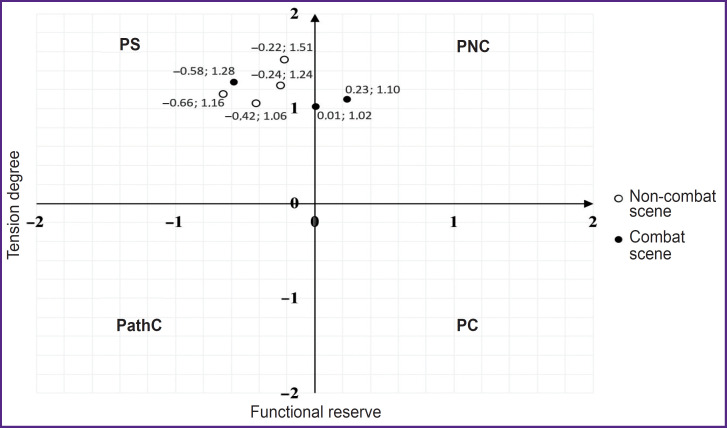
Functional state and physiological reserves dynamics when VR-content is demonstrated to a typical representative of an “inverse” type group (Patient 18 ID) PS — physiological standard, PNC — pre-nosological conditions, PC — premorbid conditions, PathC — pathological conditions

The combatants, who had functional state improvement when watching battle scenes and deterioration when being demonstrated a non-combat scene, were referred to the third type. The conditional name of the group is “inverse” type response (Figure 4).

The distinguished three types (“anxious”, “neutral”, and “inverse”) of combatants’ response on the presented content were not unexpected and correspond to the established regularities of clustering both physiological [16] and psychophysiological [17] response models (variants) on standardized stimulation conditions. It can be due to patient’s ontogenetic factors [18] and individual characteristics, job-related and life experience [19, 20] including battle experience [21], as well as the intensity of PTSD symptoms [22, 23].

The mathematical model of functional states uses the indices of tension degree of the regulatory systems and their functional reserve calculated according to HRV analysis. The estimation technique of adaptation risks developed by Baevskiy et al. [10] is an efficient tool to study prior to and after rehabilitation periods. However, to monitor dynamic physiological parameters it is reasonable to use the methods able to work under nonstationary (e.g. outpatient) conditions that enables to efficiently assess the patient’s state changes, especially under stress.

The design index — card stress (CS) developed by Bozhokin [12] shows HRV changes on exertion compared to the indices at rest enabling to classify functional states by tension degree of the regulatory systems. The index advantages over other processing techniques of variation pulsograms are due to the increased tolerance to sharp changes of RR intervals duration under the influence of various psychophysiological contexts.

**Figure 5.**
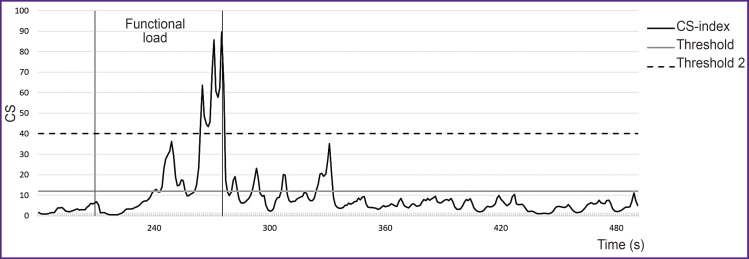
Experimental curve appearance demonstrating the dynamics of CS-index on exertion (recording from Callibri sensor)

Using CS-index enables in real-time mode to monitor the dynamics of stress load tolerance in nonstationary conditions (Figure 5). According to the study [12], the threshold values to differentiate stress tolerance levels are the following: high tolerance — up to 12, moderate — 12–40, low — over 40.

For the purpose of simplifying a visual interpretation, in the present study CS-index is smoothed out using a moving average method (window width — 20 s, step — 1 s).

**Figure 6.**
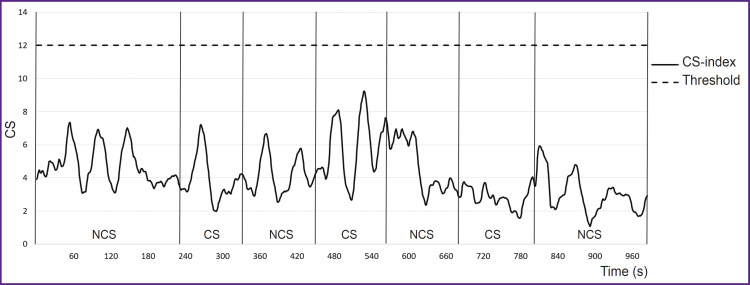
No stress reaction (Patient 31 ID) NCS — non-combat scene; CS — combat scene

**Figure 7.**
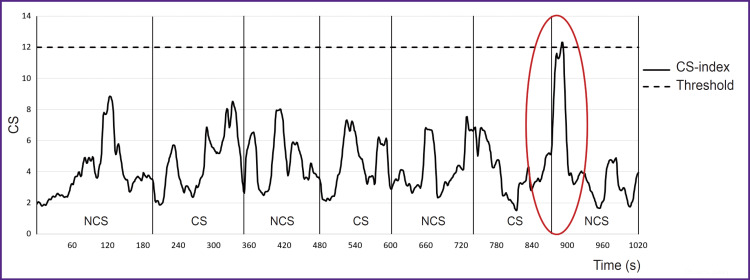
Delayed reaction (appeared after combat scenario finished, when a non-combat scenario started) (Patient 30 ID) NCS — non-combat scene; CS — combat scene

According to CS-index analysis, there were established different tolerance levels to a stress load in combatants. No threshold elevation (CS<12) indicates low tension degree of the regulatory systems (Figure 6). If the threshold value increases (CS>12), it is moderate or high tolerance level to stressors (Figure 7).

More thorough analysis of the revealed response modes and the mechanisms providing them is the study object for our investigations being carried out. However, it can be said that combat scenes provoke stress responses of different degree in combatants that is due to their individual experience, psychological trauma age, adaptivity of regulatory systems and other factors. We consider CS-index in the future to be able to determine what combat scenes demonstrated in VRET are trigger ones to form an acute stress response in a certain combatant.

### Study limitations

The present study limitations are due to the research novelty: the measurements in combatants were taken using VR, when an original content with battle scenes was demonstrated.

The methods we used to estimate the autonomic regulation dynamics were previously approved only on healthy people sampling: Baevskiy indices (FR and TD) — on astronauts with the following adaptation on larger sampling of other cohorts under study [14]; CS-index by Bozhokin — on healthy volunteers including operators and athletes [12]. The fact that these methods were not initially designed to estimate combatants’ functional state and stress responses necessitates to develop a clear measurement protocol and a reasoned data interpretation, as well as the larger sampling: both combatants and controls keeping to inclusion and exclusion criteria.

We tested the feasibility of using FR and TD indices to assess an individual type of patient’s autonomic regulation and the condition of his regulatory systems at the study entry. CS-index was used for an online assessment of the functional state dynamics during VR stimulation in order to timely detect stress episodes. It should be noted that FR and TD indices can have no correlation with a response to a trigger event determined by CS-index. It also requires additional research.

The variety of individual reactions is due to personal experience (both: in battles, and civil life) and individual psychophysiological peculiarities of a person. Accordingly, the term “trigger event” is to be defined; we use the term to mean a piece of VR-content causing a stress response in a combatant. An engram related to a traumatic event can be triggered by both: a combat and a non-combat scene, which may be associated, for example, with the unreliability of a specific type of cover or with the subjective perception of the deceptive nature of silence. In addition, response types in some patients can manifest inconsistently. This fact eventually can result in the correction of our preliminary classification of response types.

There are plan to develop a therapeutic model with a protocol of using VRET as a course including 10 sessions. We consider it to help correct the data analysis and significantly improve the understanding of PTSD formation mechanisms in combatants.

## Conclusion

The hardware and software system under development is an original domestic technology combining the possibility of simultaneous usage of exposure therapy and virtual reality to rehabilitate combatants with post-traumatic stress disorder. The suggested methodology enables to continuously monitor psychophysiological parameters during a certain session, and analyze their dynamics throughout a therapy course.

Using several stress markers gives the possibility to classify combatants by their adaptive potential at the beginning and at the end of a course of exposure therapy using virtual reality (Baevskiy approach); use online control of a patient functional state in virtual environment, and create conditions for controlled information influence (CS-index by Bozhokin).

As a result of the present study, there were revealed three main response variants: “anxious”, “neutral”, and “inverse”. In the future it will enable to choose a personalized program of rehabilitation measures for each response type using the hardware and software system. Biological feedback on heart rate variability included in the hardware and software system will help develop and consolidate in a patient the skill of independent correction of his condition with a focus on the dynamics of the recorded individual indicators.
